# Moderate Treadmill Exercise Training Improves Cardiovascular and Nitrergic Response and Resistance to *Trypanosoma cruzi* Infection in Mice

**DOI:** 10.3389/fphys.2017.00315

**Published:** 2017-05-18

**Authors:** Bruno F. C. Lucchetti, Nágela G. Zanluqui, Hiviny de Ataides Raquel, Maria I. Lovo-Martins, Vera L. H. Tatakihara, Mônica de Oliveira Belém, Lisete C. Michelini, Eduardo J. de Almeida Araújo, Phileno Pinge-Filho, Marli C. Martins-Pinge

**Affiliations:** ^1^Department of Physiological Sciences, Center of Biological Sciences, State University of LondrinaLondrina, Brazil; ^2^Department of Pathological Sciences, Center of Biological Sciences, State University of LondrinaLondrina, Brazil; ^3^Department of Histology, Center of Biological Sciences, State University of LondrinaLondrina, Brazil; ^4^Department of Physiology and Biophysics, Institute of Biomedical Sciences, University of Sao PauloSao Paulo, Brazil

**Keywords:** Chagas disease, exercise training, nitric oxide, arterial pressure, heart rate

## Abstract

There is evidence suggesting that exercise training (ET) acts as a factor toward resistance to *Trypanosoma cruzi* infection. However, the effects of mean arterial pressure (MAP), heart rate (HR), and nitric oxide (NO) during the acute phase of infection has not been elucidated yet. Swiss mice were randomly assigned into four groups: sedentary control (SC, *n* = 30), trained control (TC, *n* = 30), sedentary infected (SI, *n* = 30), and trained infected (TI, *n* = 30). ET was performed on the treadmill for 9 weeks. After training, the mice were infected with 5 × 10^3^ trypomastigotes of *T. cruzi* (Y strain) or PBS. We observed resting bradycardia and improved performance in trained animals compared with sedentary ones. On the 20th day post-infection (DPI), we found a decrease in HR in SI animals compared to TI animals (699.73 ± 42.37 vs. 742.11 ± 25.35 bpm, respectively, *P* < 0.05). We also observed increased production of NO in cardiac tissue on the 20th DPI in the SI group, normalized in TI group (20.73 ± 2.74 vs. 6.51 ± 1.19 μM, respectively). Plasma pro-inflammatory cytokines (IL-12, TNF-α, IFN-γ,) and MCP-1 were increased in SI animals, but decreased in TI animals. The increase in parasitemia on the 15th and 17th DPI in the SI group was attenuated in the TI group. Our results suggest that previous ET plays a preventive role in resistance to *T. cruzi* infection, modulating cardiovascular aspects, inflammatory reaction, and NO levels of infected mice.

## Introduction

Chagas disease (CD) is a complex disease caused by infection with the protozoan parasite *Trypanosoma cruzi* (Chagas, 1909). CD is endemic in Latin America but is increasingly found in other parts of the world, including countries previously considered free of CD such as Japan (Imai et al., 2015), Australia, and New Zealand (Jackson et al., 2014; Pinto et al., 2014). There is an estimate of 6–7 million people currently infected with *T. cruzi*, and the cost of treatment for CD remains substantial. Since the discovery of *T. cruzi* as the etiological agent of CD, a cure has been pursued, but no improvements in chemotherapy have been made regarding safety or effectiveness, despite a half century of intense research (Gaspar et al., 2015).

Clinically, *T. cruzi* infection causes acute myocarditis, followed by chronic cardiomyopathy, in humans and experimental models (Scharfstein and Andrade, 2011; Henao-Martínez et al., 2015). Most infected individuals remain asymptomatic for several years. According to older studies about 20–30% of them, 10–30 years after infection, will develop lesions clinically manifest, mainly in the heart with a presentation that is usually mild (Clayton, 2010; Coura and Borges-Pereira, 2010; Rassi et al., 2010; Morillo, 2013).

Moderate physical exercise (ET) contribute to the preservation of nitrergic (Moreira et al., 2014b) and colonic myenteric (Moreira et al., 2013, 2014a) neurons during *T. cruzi* infection. Pre-infection ET induced some improvement toward the immune response to *T. cruzi* infection in mice (Schebeleski-Soares et al., 2009) and rats (Novaes et al., 2016). ET also contributes a factor of resistance against the development of infections in animals (Moreira et al., 2013, 2014a,b) by stimulating an immune response (Nagatomi, 2006; Schebeleski-Soares et al., 2009). Mice infected with *T. cruzi* develop a cardio/anemic/renal syndrome in the acute phase, and on the 15th day post infection (DPI), the arterial pressure increases above basal levels in BALB/c mice (Oliveira et al., 2009). On the other hand, ET is recognized as a hypotensive agent, also acting as a powerful stimulus for cardiovascular structural remodeling (Kokkinos et al., 2001; Chrysohoou et al., 2003; Morvan et al., 2013).

The pro-inflammatory cytokines IL-12, IFN-γ, and TNF-α act in concert to activate macrophages to kill parasites such as *T. cruzi* through the production of nitric oxide (NO) and nitrogen free radicals (Vespa et al., 1994; Machado et al., 2012). It is known that while enhancing intracellular parasite killing NO may also contribute to damage of the host tissue, including the heart, resulting in acute myocarditis and chronic cardiomyopathy (Perez-Fuentes et al., 2003; da Matta Guedes et al., 2010). NO is an important cytotoxic and cytostatic factor in cell-mediated immunity to intracellular pathogens (Brunet, 2001), and it excessive production may cause host injury, including a reduction in myocardial contractibility (Elahi et al., 2007). At the same time, NO is a recognized cardiac and vascular mediator that influences the mechanisms of cardiovascular regulation through effects on heart rate and peripheral vascular resistance (Ghimire et al., 2017). However, the cardiovascular effects of *T. cruzi* infection, as well as changes in plasma and cardiac nitric oxide after previous ET have not been addressed thoroughly.

## Materials and methods

### Ethics statement

This study was performed in strict accordance with the recommendations in the Guide for the Care and Use of Laboratory Animals of the Brazilian National Council of Animal Experimentation and the Federal Law 11.794 (10/2008). The institutional Scientific Commission and the Committee for Animal Ethics of State University of Londrina (CEUA/UEL: 28105.2014.72) approved all the procedures used in the present study.

### Moderate physical exercise protocol

The treadmill training was performed on a low-speed, motor-driven rodent treadmill (KT-300 W, Inbramed, Porto Alegre, Brazil). For exercise adaptation, all mice were conditioned at 8 m/min, 10 min/day, for 5 days to become familiar with the treadmill environment. After this period, the treadmill ET was performed according to Ferreira and collaborators (Ferreira et al., 2007) for 9 weeks. The room temperature was maintained at 20–22°C during the training sessions. This physical exercise protocol is considered to require light or mild effort. The sedentary animal group remained in their home cage throughout the course of the experiment.

### Animals, parasites, and experimental *T. cruzi* infection

Swiss male mice purchased from the Animal House of the State University of Londrina, Parana, Brazil, were used in this study. Mice were housed in standard clear plastic cages and kept at 21°C, with free access to food and water, and a light/dark cycle of 12:12 h. Commercial rodent diet (Nuvilab-CR1, Quimtia-Nuvital, Colombo, Brazil) and sterilized water were provided *ad libitum*. The Swiss mice were randomly assigned into four groups (*n* = 30/group): sedentary control (SC), trained control (TC), sedentary infected (SI), and trained infected (TI). After the treadmill ET, experimental group mice were infected with 5 × 10^3^ trypomastigotes forms of Y strain (Type I) of *T. cruzi* (Silva and Nussenzweig, 1953). Control group mice received PBS (phosphate-buffered saline, pH 7.2). Parasites were obtained from the blood of infected mice, washed three times with phosphate-buffered saline by centrifugation, and inoculated intraperitoneally. For the analyses we have to euthanize at 7, 14, and 20 days post-infection.

### Parasitemia and survival rates

Parasitemia curve and parasite peak were determined by collecting 5 μL of blood samples from the tail of the animal, as described by Brener (1962). The blood was collected daily beginning from the 3rd DPI until no parasites were observed (approximately 30 DPI), characterizing the acute phase of infection. Survival rates of infected sedentary and trained groups of mice were evaluated daily until 60 DPI. After inducing the infection, the animals were monitored every 8 h until the 60th DPI. The animals of the infected groups utilized in this study died spontaneously (sudden death), so it was not possible to use humane endpoints because these animals did not show the signs indicating moribund conditions as changes in behavior (low activity, abnormal aggression and continuous licking of body), frequency of feeding (decreasing food or water consumption) or piloerection. The surviving animals were anesthetized with ketamine and xilazine and euthanized by cervical dislocation at the end of the experiment (60th DPI).

### Cardiac parasitism

On 7, 14, and 20 DPI, SI and TI mice were euthanized by cervical dislocation in accordance with Committee on the Ethics of Animal Experiments at Londrina State University (CEEA-UEL). For the euthanasia procedure, animals were anesthetized with ketamine (80 mg/kg body weight) and xylazine (5 mg/kg body weight). After that, the heart of each mouse was removed, fixed in 10% buffered formalin, and then sectioned. Segments were paraffin embedded and sections were stained with hematoxylin/eosin (H&E) and analyzed by light microscopy. The number of parasite nests was counted manually in fifty microscope fields (400 × magnification) per tissue section, in a double blind analysis. Three sections were evaluated, and the data expressed as the mean of the three sections.

### Quantification of heart-infiltrating cells

The total number of nucleated cells was counted in 50 microscopic fields in at least four representatives, nonconsecutive, HE-stained sections (7 μm thickness) from each mouse. Sections were examined using a Zeiss Integrations platte II eyepiece (Zeiss Co, Oberkochen, Germany) reticule, using a microscope at a final magnification of 1000X. Images were analyzed using the software ImageJ v1.49 (National Institute of Health, USA).

### NO quantification

NO concentration in plasma and heart tissue was obtained from SC, TC, SI, and TI groups of mice at 7, 14, and 20 DPI. NO was estimated by measuring nitrite, as described previously (Navarro-Gonzalvez et al., 1998; Panis et al., 2011). All reagents for the nitrite assay were obtained from Sigma Chemical Co.

### Level of plasma cytokines

IL-6, IL-10, monocyte chemoattractant protein-1 (MCP-1), interferon-γ (IFN-γ), tumor necrosis factor (TNF), and IL-12 were determined by cytometric bead array (BD™ CBA mouse inflammation Kit, San Jose, CA, USA). Briefly, 50-μL plasma samples were subjected to analysis in duplicate using the cytometric bead array kit on a C5 cytometry. The concentration of plasma cytokines was quantified using FCAP Array™ v. 3.01, SoftFlow©. The theoretical limits of detection were 5 pg/mL for all cytokines.

### Non-invasive cardiovascular measurements

The heart rate (HR) and mean arterial blood pressures (MAP) were measured by CODA Mouse & Rat Tail-Cuff Blood Pressure System (KENT Scientific CO., Connecticut, USA) in conscious mouse from the SC, TC, SI and, TI groups at 7, 14, 20 DPI and the mean value was calculated for each mouse.

### Statistical analysis

The results were expressed as arithmetic mean ± standard error of the mean (SEM). Comparison between groups was carried out via two-way ANOVA followed by Tukey's and/or Sidak's test, when appropriate. Survival rate was determined by Gehan-Breslow-Wilcoxon test using GraphPad Prism Software version 6.0 (GraphPad Software, San Diego, CA). Differences were considered statistically significant when *P* < 0.05.

## Results

### Treadmill exercise performance

**Figure 1:**
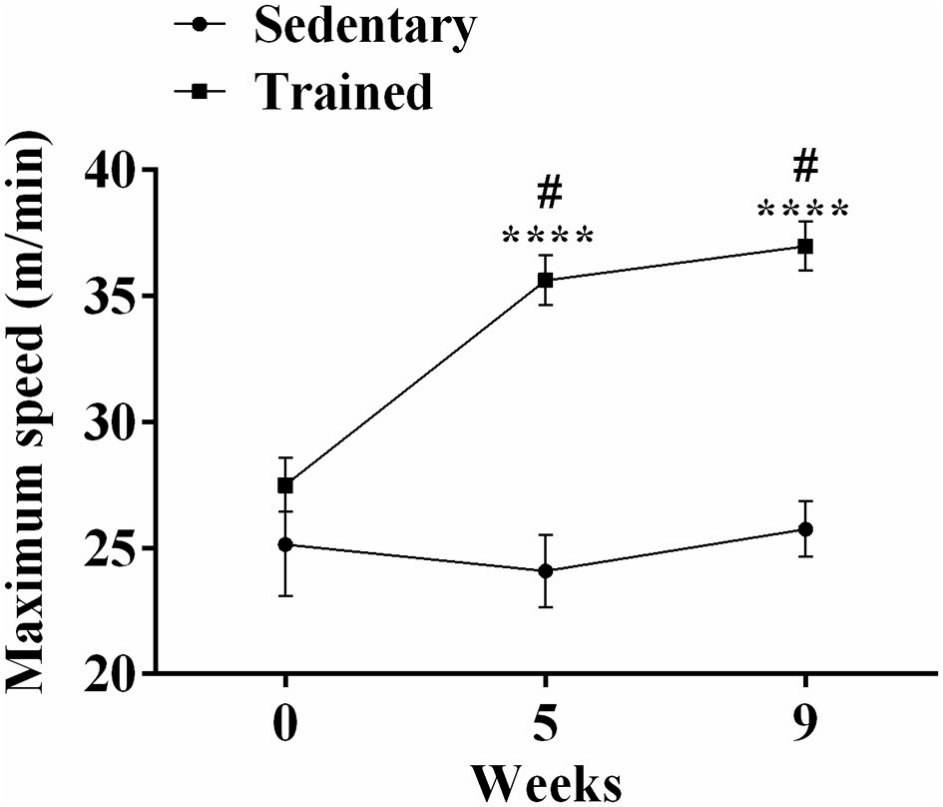
**Trained mice have significantly higher maximal speed values than sedentary mice**. The experimental groups were composed of 60 trained mice and 60 sedentary mice. ^****^P < 0.0001 comparing trained group with sedentary group for each week. ^#^*P* < 0.0001 comparing in trained group between week 0, 5, and 9.

In this analysis we compare only trained vs. sedentary animals without infection. As shown in Figure 1, initially (week 0) the exercise performance test showed no significant difference between sedentary (25.15 ± 2.06 m/min) and trained (27.50 ± 1.06 m/min) mice. At the end of week 5 and 9, trained animals performed better than animals of the sedentary group (35.62 ± 0.99 vs. 24.09 ± 1.42 m/min, *P* < 0.0001; 36.97 vs. 25.75 ± 1.10 m/min, *P* < 0.0001, respectively). When we analyzed the results obtained in the 9 weeks; no statistical difference was observed in the performance of sedentary mice during this period (Figure 1). However, trained mice had an improvement in performance (27.5 ± 1.06 m/min to 35.62 ± 0.99 m/min between week 0 and 5), reaching a maximum speed of 36.97 ± 0.97 m/min (week 9, *P* < 0.0001).

### Cardiovascular parameters during exercise training

**Figure 2:**
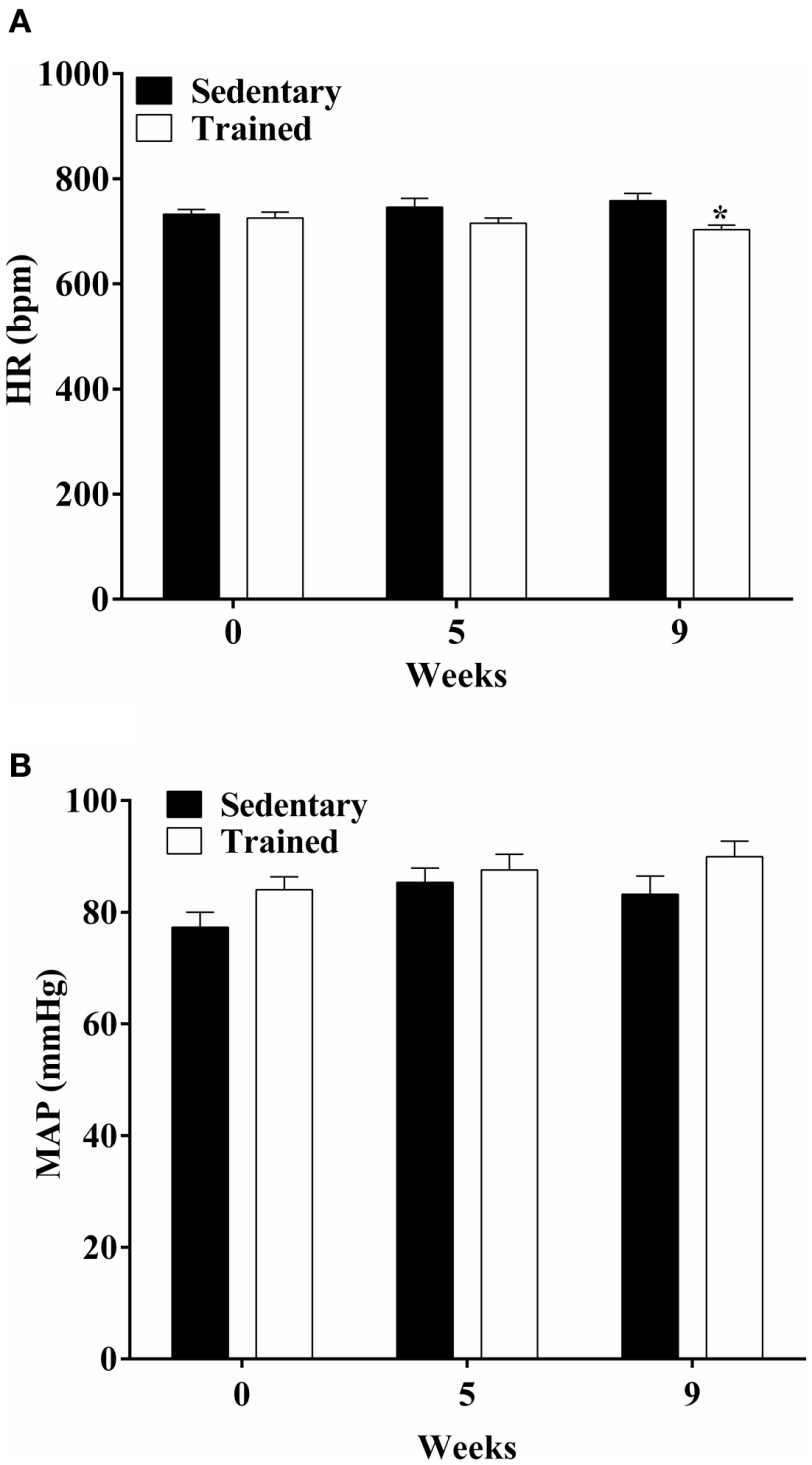
**Exercise training induces decrease in heart rate (HR) in the trained group compared to their respective sedentary group. (A)** HR, **(B)** mean arterial pressure (MAP) at 0, 5, and 9 weeks of treadmill exercise training. No changes were observed in MAP of the trained group when compared to the sedentary group. The experimental groups were composed of 27 trained mice and 14 sedentary mice. ^*^P < 0.05 comparing trained with sedentary at week 9.

In this analysis we compare trained vs. sedentary animals before infection. ET induced resting bradycardia was observed in the trained groups compared to their respective sedentary groups (week 9, Figure 2A, *P* < 0.05). No changes were observed in the mean arterial pressure (MAP) in the trained group when compared to the sedentary group (Figure 2B).

### Effect of treadmill exercises on course of *T. cruzi* infection

**Figure 3:**
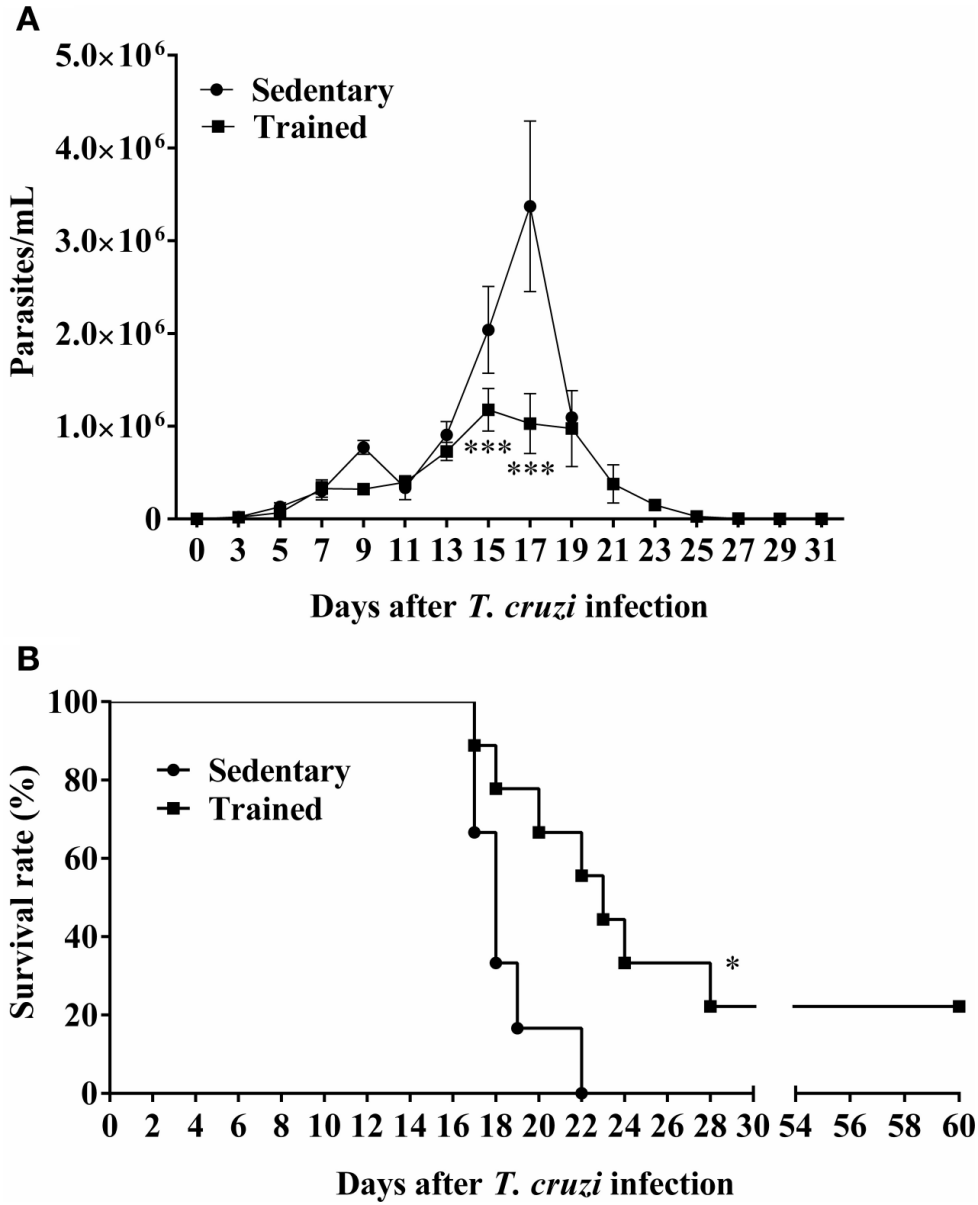
**Effect of treadmill exercises on the course of *T. cruzi* infection in mice**. Parasitemia and survival were determined in Swiss mice infected with 5 × 10^3^ blood trypomastigotes *T. cruzi* (Y strain) 2 days after the end of the physical activity protocol. **(A)** Parasitemia was quantified as trypomastigotes per milliliter of blood. The data (mean ± SEM) are representative of two independent experiments. ^***^P < 0.001 comparing trained vs. sedentary mice. **(B)** Survival rate was determined by Gehan-Breslow-Wilcoxon test. The number of animals of the experimental groups was 9 trained and 6 sedentary. ^*^P < 0.05 comparing trained mice with sedentary mice.

In this analysis we compare trained vs. sedentary animals after infection only. The parasitemia curve showed a characteristic profile of the Y strain of *T. cruzi* in the two infected groups. Thirteen days following inoculation with *T. cruzi*, parasitemia was similar between the two groups. The parasite peaks occurred on 15 and 17 DPI and were lower in the trained group compared to the sedentary group (Figure 3A, *P* < 0.05 to 15 and 17 DPI, *P* < 0.001). There were significant differences in survival between the two groups (Figure 3B, *P* < 0.05). It was observed that none of the animals in the sedentary group survives after 22 DPI, with mortality beginning at 17 DPI, while 22% of all mice in the trained group survive until 60 DPI (Figure 3B).

### Effects of treadmill exercise on parasitism and inflammatory infiltrate in the heart of infected mice

**Figure 4:**
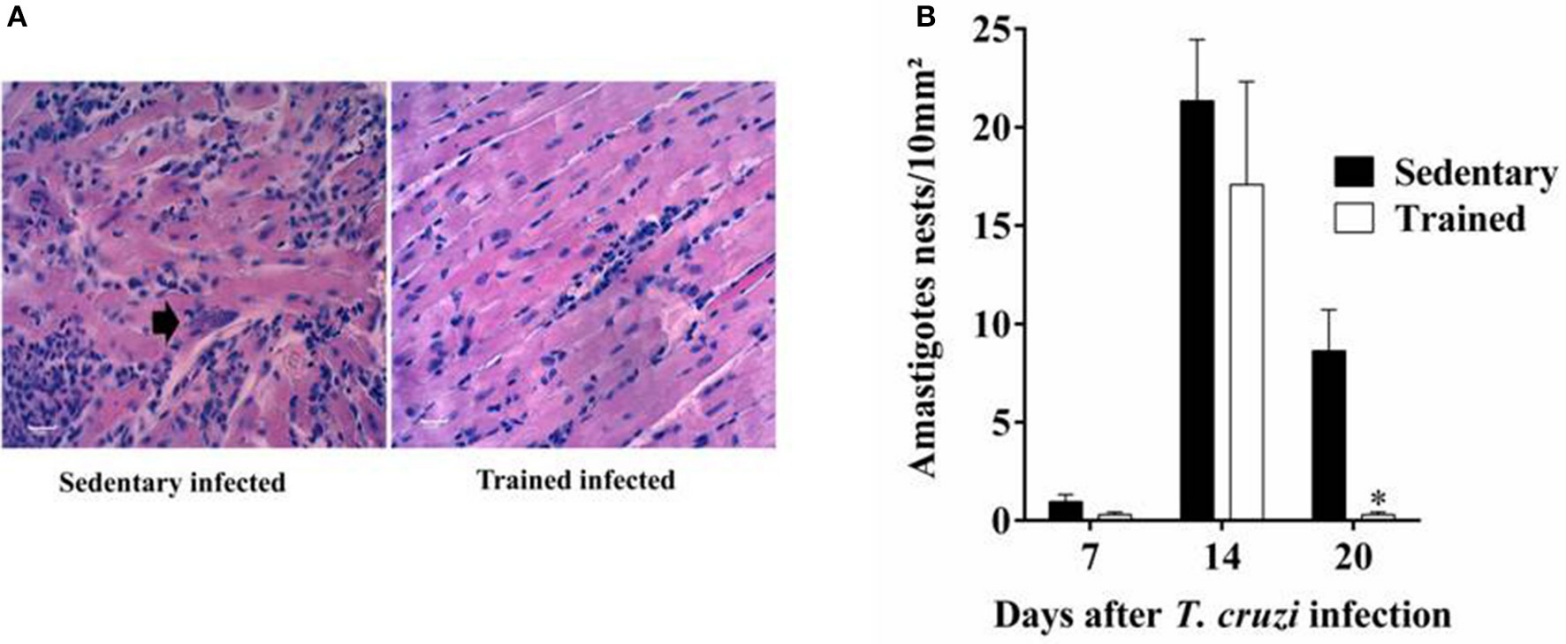
**Treadmill exercises control parasite proliferation in the heart**. Swiss mice infected with *T. cruzi* were sacrificed on 7, 14, and 20 days post-infection (DPI) and tissue parasitism in the heart evaluated as described in material and methods. **(A)** Representative microphotographs (original magnification X 400) of cardiac tissue of mice at 20 DPI is shown. **(B)** Bars represent mean ± SEM of five animals per group. ^*^P < 0.05 comparing trained and sedentary animals at 20 DPI.

In this analysis we compare trained vs. sedentary animals after infection only. As expected, *T. cruzi* infection increased the cardiac parasitism in heart tissue of mice (Figures 4A,B). There was a significant reduction in cardiac parasitism (20 DPI, Figure 4B, *P* < 0.05) of trained mice when compared with sedentary mice.

**Figure 5:**
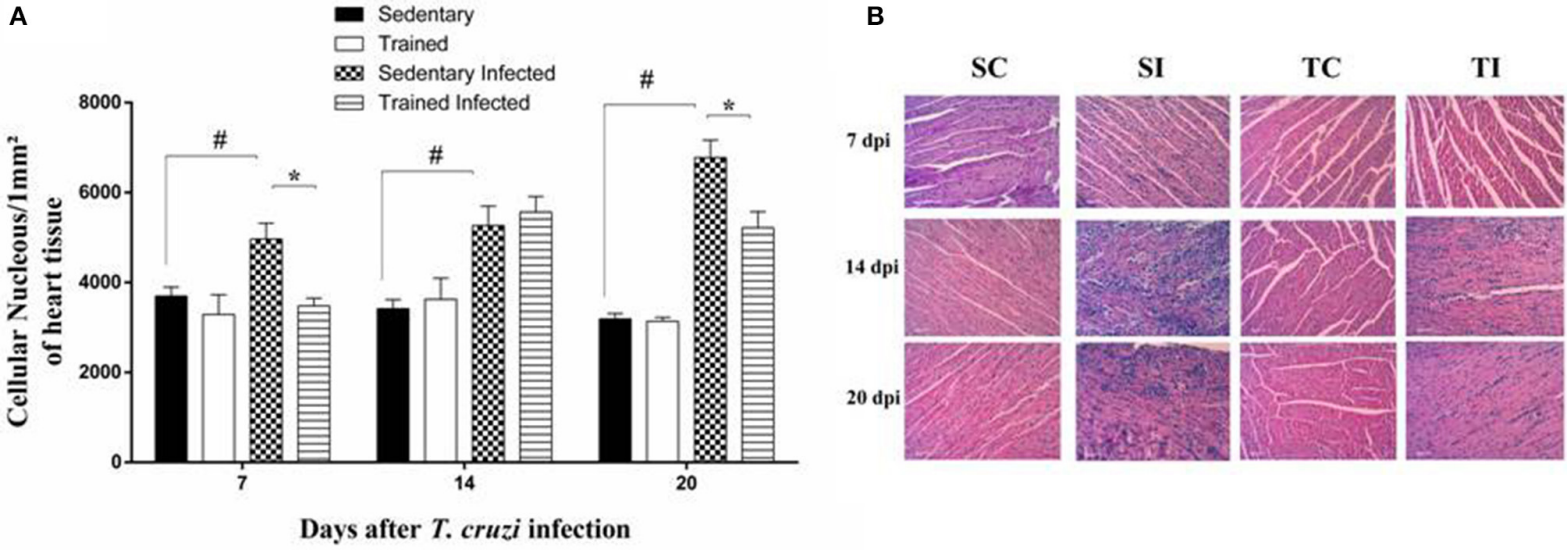
**Treadmill exercise decreases *T. cruzi*-induced heart pathology. (A)** Quantification of nuclei in the heart tissue of uninfected sedentary and trained mice and infected sedentary and trained mice 7,14, and 20 days post-infection (DPI). **(B)** Representative microphotographs (original magnification X 400) of cardiac tissues of mice at 7, 14, and 20 DPI are shown. Data (mean ± SEM) are representative of two independent experiments using five mice per group. ^*^P < 0.05 comparing sedentary-infected mice with trained infected mice. ^#^*P* < 0.05 comparing sedentary infected with sedentary uninfected mice. Days post infection (DPI), Sedentary control (SC), trained control (TC), sedentary infected (SI), and trained infected (TI).

The total number of nuclei per 1 mm^2^ section of heart tissue was determined in all experimental groups of animals. *Trypanosoma cruzi* infection increased the number of inflammatory cells in the heart tissue of sedentary mice at 7 DPI (3,697.30 ± 200.40 vs. 4,964.60 ± 355.90 *P* < 0.05), 14 DPI (3,419.00 ± 200.30 vs. 5,265.70 ± 433.75, *P* = 0.003), and 20 DPI (3,191.10 ± 119.74 vs. 6,776.40 ± 388.37, *P* < 0.001) (Figure 5A) when compared with sedentary uninfected mice.

The ET decreased the number of nuclei per section of heart tissue at 7 and 20 DPI when compared with sedentary-infected mice (7DPI: 6,776.40 ± 388.37 vs. 4,964.60 ± 355.90, *P* < 0.05; 20 DPI: 5,219.60 ± 356.18 vs. 3,481.60 ± 168.69, *P* < 0.05; Figure 5A).

It is noteworthy that by microscopic analysis, the inflammatory infiltrates found in all infected mice was characterized by mononuclear cells and the scarce presence of polymorphonuclear cells. Furthermore, no differences were detected in the cellular composition of the myocarditis between the infected mice at 14 DPI (Figure 5B).

### Effects of treadmill exercise on cardiovascular parameters during infection

**Figure 6:**
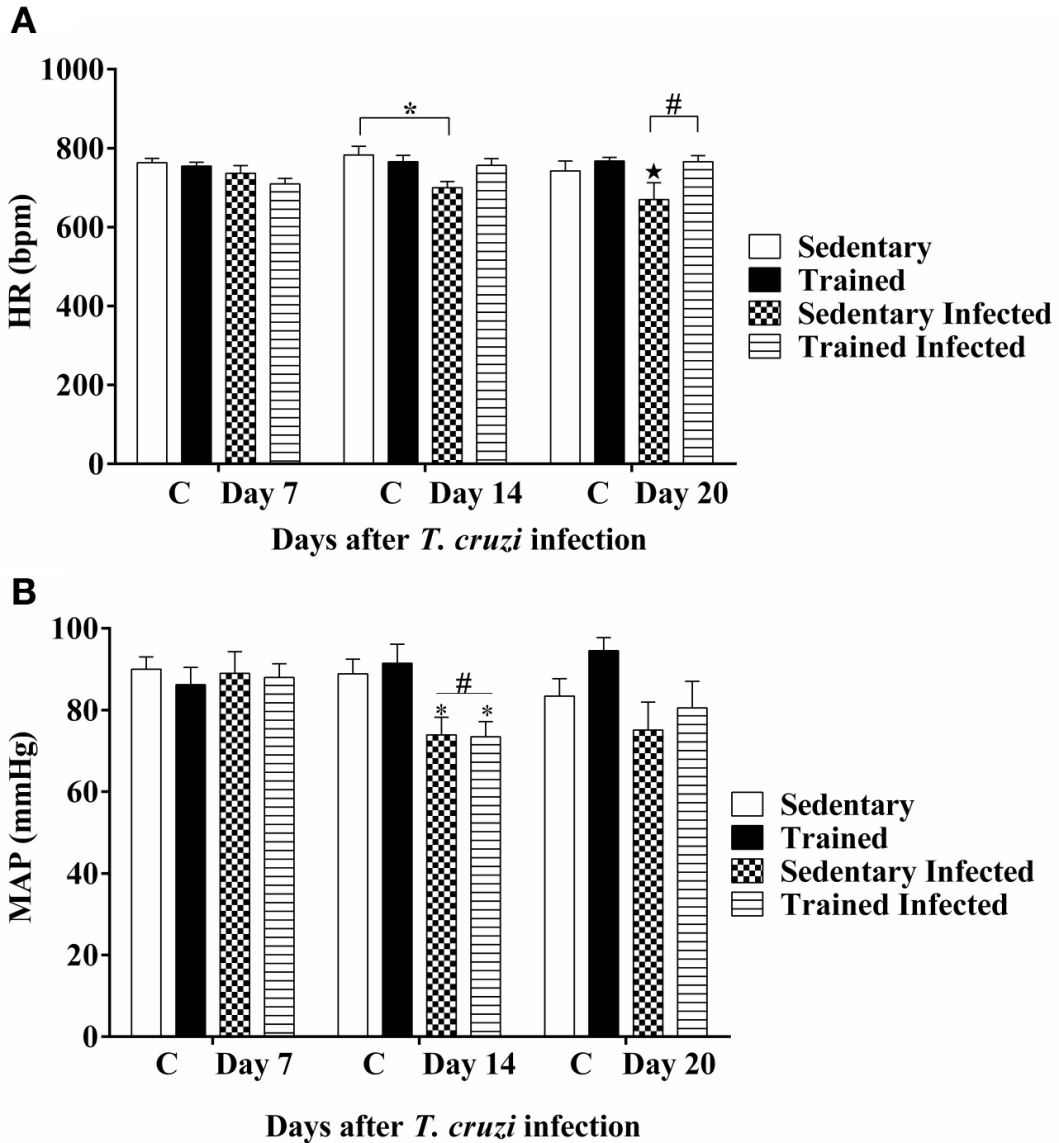
**Effect of exercise training on cardiovascular parameters after *T. cruzi* infection. (A)** Heart rate (HR), ^*^P < 0.05 comparing sedentary not-infected with sedentary infected (14 DPI); ^⋆^*P* < 0.05 comparing sedentary infected (20 DPI) with sedentary infected (7 DPI) and ^#^*P* < 0.05 comparing sedentary infected (20 DPI) with trained infected (20 DPI). **(B)** Mean arterial pressure. *P < 0.05 comparing sedentary infected (14 DPI) and trained infected (14 DPI) with sedentary infected (7 DPI) and trained infected (7 DPI); ^#^*P* < 0.05 comparing sedentary infected (14 DPI) and trained infected (14 DPI) with non-infected and age matched control mice. Days post infection (DPI), 10 mice per group.

In evaluating the cardiovascular parameters post infection, we observed that HR remained the same in all groups 7 DPI. However, at 14 DPI, the sedentary infected animals presented bradycardia compared to sedentary uninfected mice (699.42 ± 15.93 vs. 782.95 ± 22.30 bpm, *P* < 0.05). At 20 DPI, we observed that the average HR of sedentary infected mice remained lower compared to that in trained infected mice (699.73 ± 42.37 vs. 742.11 ± 25.35 bpm, *P* < 0.05). However, trained-infected mice did not present bradycardia at 14 and 20 DPI, similar to non-infected mice (Figure 6A).

We observed no statistical difference in MAP between groups of animals at 7 DPI. However, at 14 DPI we observed a decrease in MAP in infected animals compared to their respective control animals (sedentary: 73.92 ± 4.28 vs. 88.85 ± 3.60 mmHg, *P* < 0.05; and trained: 73.45 ± 3.69 vs. 91.48 ± 4.67 mmHg, *P* < 0.05). The MAP of infected animals was lower compared to the first evaluation at 7 DPI. At 20 DPI, we did not find a statistical difference in MAP between groups. However, there was a slight recovery in the most marked hypotension in the trained and infected animals without presenting statistical difference (Figure 6B).

### Nitric oxide production

**Figure 7:**
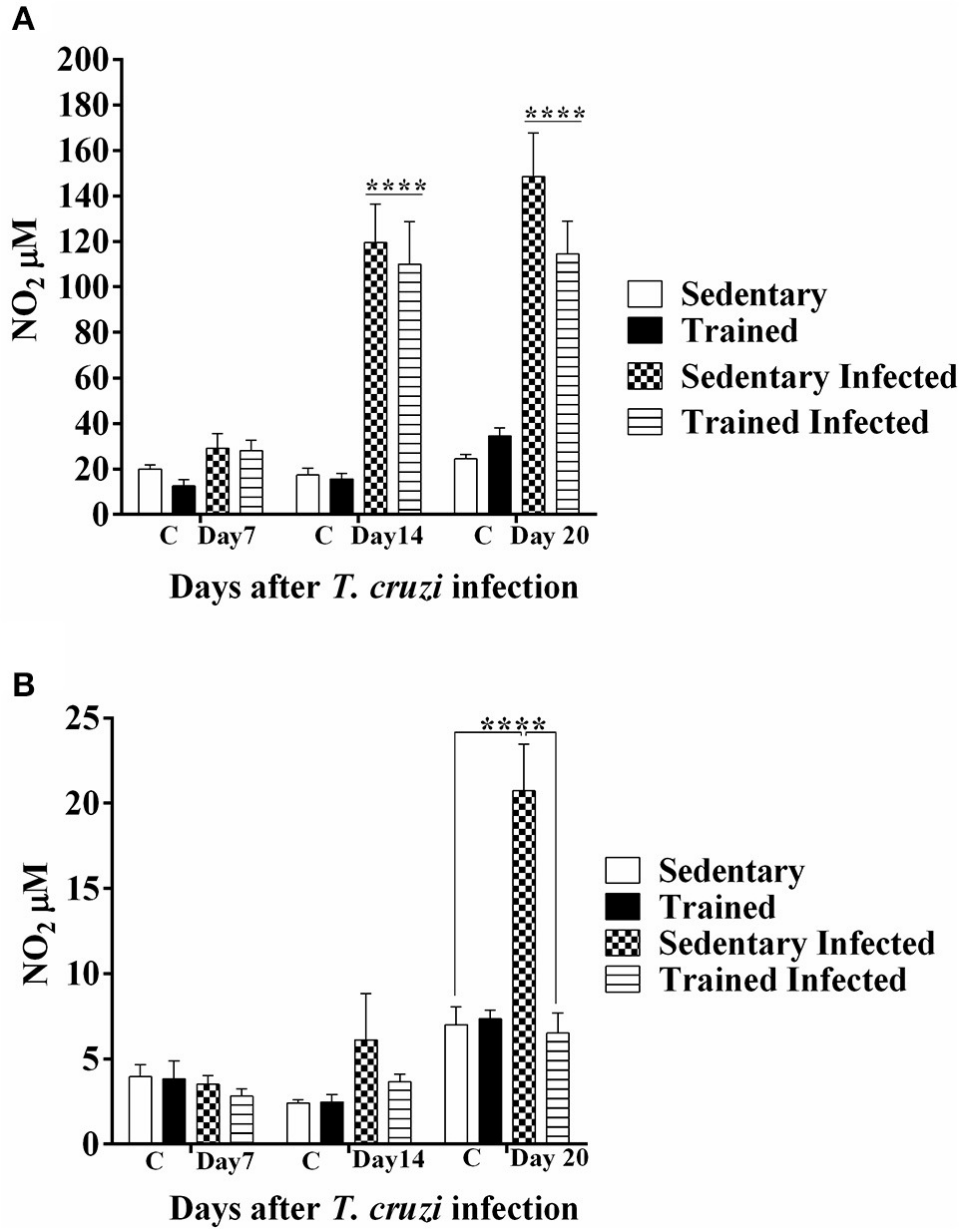
**Treadmill exercise modulates nitric oxide (NO) production**. Sedentary and trained mice infected or not with *T. cruzi* were bled at 7, 14, and 20 days post-infection (DPI) for NO measurements. NO was estimated by measuring nitrite levels via the cadmium–copper system followed by Griess reaction, as described in material and methods. **(A)** Levels of NO in the plasma. ^****^P < 0.0001 comparing infected mice (14 and 20 DPI) with infected (7 DPI) and non-infected age matched control mice, five mice per group. **(B)** Levels of NO in the heart. *P* < 0.0001 comparing sedentary infected with sedentary uninfected and trained infected mice.

Compared to control groups (not infected), the plasma NO levels of acutely (14–20 DPI) infected mice were significantly increased (Figure 7A, *P* < 0.0001). No change in plasma NO levels were observed in trained-infected mice compared to sedentary-infected mice (*P* > 0.05, Figure 7A). However, the NO levels in the heart of sedentary-infected mice were higher than in uninfected mice (20 DPI, Figure 7B, *P* < 0.0001). The NO levels in the heart of trained-infected mice decreased vs. the sedentary infected group (20 DPI, *P* < 0.0001, Figure 7B).

### Plasma cytokines

**Figure 8:**
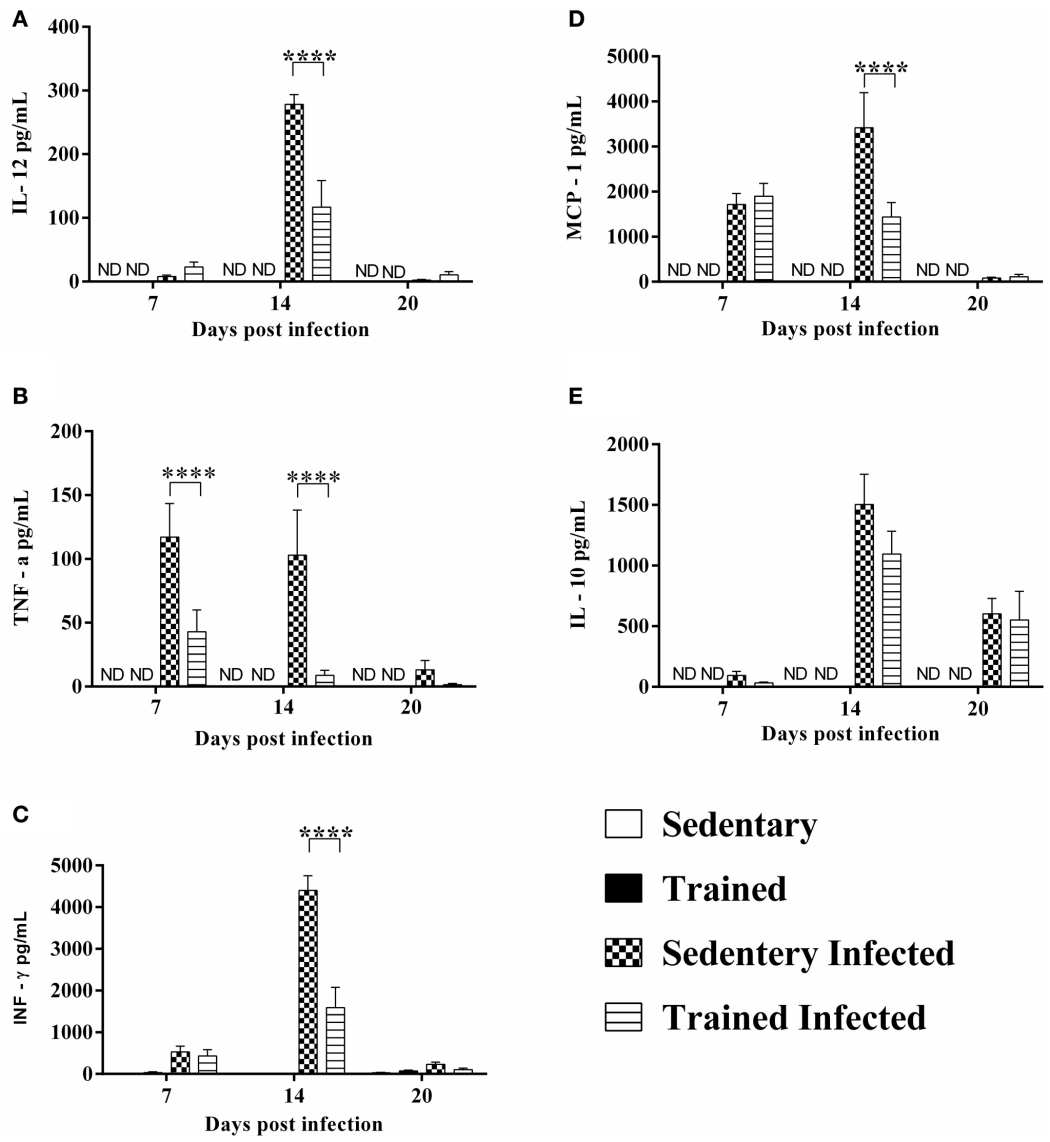
**Effect of treadmill exercises on pro-inflammatory cytokines in infected mice. (A)** IL-12, **(B)** TNF-α, **(C)** INF-γ, **(D)** MCP-1, and **(E)** IL-10 plasma levels were quantified using the BD CBA mouse inflammation kit for a flow cytometer. The data show the mean ± SEM of five mice per group and are representative of two independent experiments. ^****^P < 0.0001 comparing sedentary infected with trained infected.

The analysis was determined in all experimental groups of animals. A significant increase of pro-inflammatory cytokines (IL-12, TNF-α, IFN-γ, and IL-10) and MCP-1 was observed in the plasma of sedentary-infected mice (*P* < 0.0001, Figure 8). Moreover, IL-6 was not detectable in plasma of mice from control and infected groups (data not shown). A significant decrease of IL-12 (14DPI), TNF-α (7 and 14DPI), IFN-γ (14DPI), and MCP-1 (14DPI) in plasma was observed in a trained-infected group vs. the sedentary infected group (*P* < 0.0001, Figures 8A–D). However, no difference was observed for IL-10 (Figure 8E, *P* > 0.05).

## Discussion

The results of the present study suggest that previous ET plays a preventive role in resistance against *T. cruzi* infection by modulating the inflammatory reaction and NO levels of infected mice. Furthermore, changes in HR and arterial pressure during the acute phase of *T. cruzi* infection, as the nitric oxide levels in cardiac tissue and plasma, were observed, and have not been previously described yet. Our data collaborate to elucidate the effects of nitric oxide, and inflammatory mediators on cardiovascular manifestations during acute experimental Chagas disease, as the benefits of regular previous exercise.

The treadmill exercise protocol used in this study improved the performance of the trained mice compared with the sedentary, corroborating the results obtained by Hoydal et al. (2007). In fact, moderate physical activity is correlated with positive effects on the cardiovascular system (Martins-Pinge, 2011). It provides several cardiovascular benefits including the refurbishment of the heart and skeletal muscle (DiCarlo and Bishop, 1990; Mack et al., 1991), and improvements in the autonomic control of the heart (Brum et al., 2000). Furthermore, the appearance of resting bradycardia is a characteristic marker of ET (Negrao et al., 1993; Brum et al., 2000), which was also found in our work at the end of the 9th week of training. The benefits to the cardiovascular system via central and peripheral mechanisms in humans and animals are also evident from the action of moderate physical activity (Fernandes and Zanesco, 2010; Martins-Pinge, 2011).

It is also known that the duration and intensity of the physical activity can be determinant to induce or not the physiological adaptations to the cardiovascular system (Forjaz et al., 1998). Moderate intensity and long duration exercises, such as swimming and running, have been shown to be efficient non-pharmacological therapies in promoting cardiovascular health by increasing systolic volume (Melo et al., 2003), capillary angiogenesis (Amaral and Michelini, 2011), bradycardia (Mastelari et al., 2011; Raquel Hde et al., 2016), cardiac hypertrophy (Souza et al., 2007) and baroreflex gain (Brum et al., 2000). Due to the positive effects of the running for cardiovascular rehabilitation, treadmill physical training protocols have been applied as allies in the treatment of arterial hypertension (Brum et al., 2000) (Veras-Silva et al., 1997) and heart failure (Krieger et al., 2001; Ichige et al., 2016), among others.

The average blood pressure of trained and sedentary mice remained unchanged throughout the physical training protocol, corroborating the findings of other authors (Negrao et al., 1992; De Angelis et al., 1997, 2004). However, during infection we observed that sedentary mice presented a stable control of arterial pressure variation as infection progressed, but exhibited dramatic oscillations of HR after peak parasitemia. The MAP of sedentary- and trained-infected mice dropped from levels ranging 80–90 mmHg at 7 DPI to 70–80 mmHg at 14 DPI, further decreasing as infection progressed, while also coinciding with increasing NO levels in the plasma. These results suggest that NO may be responsible for the arterial pressure alterations during acute *T. cruzi* infection, considering its well-known role in vasodilation (Moncada et al., 1988). Similar results were found in another study, where high levels of NO during infection by *T. cruzi* were correlated with low blood pressures. After treatment with an iNOS inhibitor (1,400 W), the arterial pressure was normalized. However, HR was not evaluated in this study (Santiago et al., 2012).

Bradycardia is a marker of cardiovascular conditioning and was expected after moderate exercise training. However, the more pronounced bradycardia on 20th day post infection in sedentary mice may be derived from nitric oxide, as it was greater in plasma and cardiac tissue of this group of animals, and it is not related to exercise training. We believe that the origin of that increased level of NO is from iNOS source, and it contributes to the inflammatory response as literature has demonstrated during acute phase of *T. cruzi* infection. Corroborating this, data from the literature showed that in iNOS knockout mice present higher heart rate compared to control (Mani et al., 2006). Also in nNOS knockout mice it is observed an increase in heart rate due to inhibition of adrenergic-adenylate cyclase signaling within sino atrial node myocytes (Choate et al., 2008). Thus, our results suggest that the lower heart rate in trained animals may be a result of increased nNOS in the heart or central nervous system (Raquel Hde et al., 2016). On the other hand, the decrease in heart rate observed in animals during *T. cruzi* infection may be due to the increase in iNOS expression and/or activity. Further studies are necessary to demonstrate those hypotheses.

An adequate production of pro-inflammatory cytokines such as IFN-γ, TNF-α, IL-1, IL-12, and IL-6 is essential for controlling infection by intracellular parasites. To resolve the *T. cruzi* infection, a balance is necessary between the immune response mediated by TH1 and TH2. In this regard, we observed that trained animals had a significant decrease of pro-inflammatory cytokines as IL-12, TNF-α, IFN-γ. We also detected an increased NO production in the heart of TI mice, which suggests a possible involvement of these molecules in the injury of the myocardial tissue. Interestingly, the ET also decreased NO level in the heart of infected mice, suggesting a protective mechanism against myocardial injury. The increased NO levels in the heart of infected mice may be responsible for the decreased HR observed at 14 and 20 DPI, which was reverted by previous ET. The source of this NO in *T. cruzi*-infected mice has been evaluated before, and may be derived from iNOS (Panis et al., 2011). We speculate that ET abrogates the iNOS activation during *T. cruzi* infection, and may increase the activity of constitutive NOS, collaborating as an anti-inflammatory agent in the heart. However, further studies are necessary to investigate this hypothesis.

The diminished production of pro-inflammatory cytokines in the trained group was associated with higher survival rates, and suggests that both NO and pro-inflammatory cytokines are involved in the fatal outcome of the sedentary-infected mice. Moreover, we observed that the ratio of IFN-γ to IL-10 was lower in trained mice than in the sedentary-infected mice (data not shown). Our results suggest that, in trained infected animals, the protective action of IFN-γ could be more effective in the presence of the antagonistic action of IL-10, reducing the inflammatory infiltration. Inflammatory infiltration was also reduced in trained-infected animals due to a decreased production of cytokines and chemokines. These results are consistent with those of Reed et al. (1994), who observed a lower IL-10 production in resistant mice compared to the susceptible ones. Furthermore, Abrahamsohn and Coffman (1996) observed a lower number of parasites and higher IFN-γ production in IL-10 knockout (KO) mice than in the wild-type (WT) mice. However, *T. cruzi*-infected IL-10 KO mice, although having lower blood and tissue parasitism, did not survive longer, and often died slightly earlier than either infected WT or RAG KO mice. The trained mice in our study survive longer than sedentary probably due to IL-10 protection from INF-y deleterious effects (Abrahamsohn and Coffman, 1996).

Infectious microorganisms elicit acute inflammatory responses mediated by cytokines secreted from immune cells. We examined the effect of exercise training on the immune response induced by infection with *T. cruzi*. Our findings show that exercise suppresses infection-induced inflammation, as indicated by reductions in the levels in inflammatory cytokines (IL-12, TNF-α, IFN-γ,) and in another inflammation marker (NO). Production of cytokines (Zheng et al., 2015) and NO (Terra et al., 2013) is significantly increased by regular exercise, resulting in improved immune responses, and prevention of various diseases, increased signal transduction (Gjevestad et al., 2015). Secretion of large amounts of cytokines stimulated by infectious pathogens results in strong activation of immune cells. TNF-α and IL-1β, in particular, are key mediators of the signal transduction initiating immediate immune cell activity upon release from the site of infection. We observed an increase in parasitemia on 15th and 17th dpi in SI group that was attenuated in TI group. Also, TI animals showed increased survival compared to SI. So, exercise providing beneficial effects to the host by acting on the immune system to preserve its ability to exert antimicrobial and regulatory effects. These results showed that a decrease in cardiac parasitism is possible in a context of moderate inflammatory response, as occurred in TI group, which could result in a protection to cardiomyopathy.

Overall, we demonstrated that our experimental model promoted beneficial effects in the heart tissue. Histopathological evaluation showed differences in the inflammatory infiltrate, which was observed to be lower in the trained group. Similar results on the effects of physical exercise in chagasic animals were obtained in the enteric nervous system (Moreira et al., 2013, 2014a,b). The direct actions of the parasite in the cardiac muscle in the above parameters have not been previously reported in the literature. Finally, our results suggest that the application of moderate physical training had positive effects toward the prevention against *T. cruzi* infection and minimized the injury caused by the parasite, reminiscent of results to those of the uninfected control group.

In conclusion, our results show that moderate ET produced a profile of NO and cytokines different from that of the sedentary infected mice, with a delicate balance between TH1 and TH2, suitable for overcoming the infection. The positive effects of moderate aerobic ET can attend as a prophylactic action on inflammation that occurs in the early stages of *T. cruzi* infection. In our study, we were not able to introduce physical training during *T. cruzi* infection, as the animals become more debilitated and the exercise would only cause harm. However, other studies that evaluate the continuity of physical training for the chronic phase of Chagas disease will also be of great clinical relevance.

## Author contributions

Conception and design: PPF, LCM, and MCMP. Acquisition, analysis and interpretation of data: BL, HdAR, NZ, ML, VT, and MdOB. Analyzed the data: EdAA, PPF, LCM, and MCMP. Materials and reagents: EdAA, PPF, LCM, and MCMP. Drafting or revising and final approval: BL, HdAR, NZ, ML, VT, MdOB, EdAA, PPF, LCM, and MCMP.

### Conflict of interest statement

The authors declare that the research was conducted in the absence of any commercial or financial relationships that could be construed as a potential conflict of interest.
